# The study on the impact of AAA wall motion on the hemodynamics based on 4D CT image data

**DOI:** 10.3389/fbioe.2023.1103905

**Published:** 2023-03-30

**Authors:** Chen Peng, Wei He, Xingsheng Huang, Jun Ma, Tong Yuan, Yun Shi, Shengzhang Wang

**Affiliations:** ^1^ Department of Aeronautics and Astronautics, Institute of Biomechanics, Fudan University, Shanghai, China; ^2^ Department of Vascular Surgery, Zhongshan Hospital, Fudan University, Shanghai, China; ^3^ Shenzhen Raysight Intelligent Medical Technology Corporation, Shenzhen, Guangdong, China; ^4^ Institute of Vascular Surgery, Fudan University, Shanghai, China; ^5^ National Clinical Research Center for Interventional Medicine, Fudan University, Shanghai, China; ^6^ Institute of Biomedical Engineering Technology, Academy for Engineering and Technology, Fudan University, Shanghai, China; ^7^ Yiwu Research Institute, Fudan University, Yiwu, Zhejiang, China

**Keywords:** four-dimensional computed tomography angiography (4D CT), computational fluid dynamics (CFD), moving boundary method (MBM), abdominal aortic aneurysm (AAA), intraluminal thrombus (ILT)

## Abstract

**Purpose:** To analyze the effect of the physiological deformation of the vessel wall on the hemodynamics in the abdominal aortic aneurysm (AAA), this paper compared the hemodynamics in AAA based on the moving boundary (MB) simulation and the rigid wall (RW) simulation.

**Method:** Patient-specific models were reconstructed to generate mesh based on four-dimensional computed tomography angiography (4D CT) data. The dynamic mesh technique was used to achieve deformation of the vessel wall, surface mesh and volume mesh of the fluid domain were successively remeshed at each time step. Besides, another rigid wall simulation was performed. Hemodynamics obtained from these two simulations were compared.

**Results:** Flow field and wall shear stress (WSS) distribution are similar. When using the moving boundary method (MBM), mean time-averaged wall shear stress (TAWSS) is lower, mean oscillatory shear index (OSI) and mean relative residence time (RRT) are higher. When using the 10th and 20th percentile values for TAWSS and 80th and 90th percentile values for RRT, the ratios of areas with low TAWSS, high OSI and high RRT to the entire vessel wall are higher than those assuming the vessel as rigid. In addition, one overlapping region of low TAWSS, high OSI and high RRT by using the MBM is consistent with the location of thrombus obtained from the follow-up imaging data.

**Conclusion:** The hemodynamics results by using the MBM reflect a higher blood retention effect. This paper presents a potential tool to assess the risk of intraluminal thrombus (ILT) formation based on the MBM.

## 1 Introduction

Abdominal aortic aneurysm (AAA) is defined as permanent and irreversible local dilation of the abdominal aorta (Chen et al., 2014). At present, the pathogenesis of AAAs is not completely clear, but a large number of studies have shown that the occurrence of AAAs is related to degeneration in the media layer of the aorta (Salman et al., 2019). Loss of elastin, deposition and remodeling of collagen fibers lead to the formation and growth of AAAs (Gasser et al., 2006; Valentin et al., 2013). AAAs rupture when the stress acting on the vessel wall exceeds the strength of the vessel wall, and the mortality rate for ruptured AAAs patients can be 65%–85% in China (Canchi et al., 2018).

Recent studies have shown that hemodynamics plays an important role in the progression of AAAs (Tanweer et al., 2014; Boyd et al., 2016). The non-invasive method for hemodynamics simulation, which combined the clinical medical image data with the computational fluid dynamics (CFD) is needed. Notably, the hemodynamics of AAA is considered to be a key factor in the formation and growth of intraluminal thrombosis (ILT), for the ILT could prevent rupture of the AAA by reducing the stresses acting directly on the vessel wall (Arzani et al., 2014), and the prediction of ILT formation in AAAs is important.

Zambrano et al. (2016) and Gharahi et al. (2015) collected CT image data from different cases and analyzed the relationship between wall shear stress (WSS) and thrombus aggregation and AAAs growth based on the computational fluid dynamics (CFD) simulation. Doyle et al. followed up a patient for up to 2.5 years and analyzed the hemodynamics of AAA, they found that long-term low time average wall shear stress (TAWSS) promoted the AAA wall dilation and thrombus formation (Doyle et al., 2014). Suh et al. (2011a) and Suh et al. (2011b) simulated the progression of AAAs, calculated the oscillatory shear index (OSI) and particle retention time (PRT) to quantify the recirculation effects of blood in aneurysms, and found that platelet activation induced by stagnant blood flow can induce thrombus formation. However, these studies assumed the aorta as rigid and ignored the *in vivo* deformation of aorta (which is caused by blood flow, peri-arterial tissue, respiration, heartbeat, etc.)

To overcome the above shortcomings, the fluid-structure interaction (FSI) method was applied in the hemodynamics simulation in AAAs. Bluestein et al. (2009) used the FSI method to simulate the hemodynamics of two patients, with varied AAA geometries and ILT structures and compare the AAA rupture risk. Ong et al. (2018) presented evidence for one type of flow dynamics within the aneurysm sac. They performed CTA image-based patient-specific FSI modeling of three cases of aortic aneurysms, their study showed that the formation of the ILT is associated with vortex, and recirculation flow within the aneurysm sac may lead to the formation of ILT. Drewe et al. (2017) aimed to perform FSI simulations of an ideal AAA geometry to determine the influence of proximal neck and iliac bifurcation angles on AAA wall stress. They found that AAAs can expand and rupture in areas with low WSS, and large iliac bifurcation angles imply less likelihood of thrombus development.

In the above studies, the settings of the material parameters of the vessel wall were based on the previous studies that have been used by Shi et al. (2021), Shang et al. (2013), and Di Martino et al. (2001), respectively. However, for patients of different ages, the material parameters such as the thickness and elastic modulus in different regions (such as ascending aorta and abdominal aorta) are quite different (Xiong et al., 2011). Therefore, hemodynamics simulated by the FSI method may differ from physiological conditions.

The moving boundary method (MBM) can reflect the effect of the *in vivo* deformation of aorta on the hemodynamics without setting specific material parameters. Some researchers have used this method to calculate vessel wall stiffness, WSS, etc. They demonstrated the potential of MBM for clinical application (Piccinelli et al., 2013; Farzaneh et al., 2019). Danilov et al. (2017) and Lozovskiy et al. (2018) developed a stabilized finite element method and performed simulation based on 4D-CT and collected hemodynamics results of the left/right ventricle, the method of these studies is proven to be stable and robust when deformation is large, and without needing interpolation. Their work extended the application of 4D CT in hemodynamics studies. However, using the MBM to assess ILT formation risk in AAA was rarely been reported.

In this study, 4D CT image data of one AAA patient at 21 cardiac instants in one cardiac cycle were used to reconstruct the patient-specific instantaneous geometries. After mesh generation, the coordinates of each node at different time-instants were calculated. Then the user-defined function (UDF) in Fluent was used to control the movement of each node for achieving the deformation of the vessel wall, surface mesh and volume mesh of fluid domain at each time step were remeshed. After that, the hemodynamics in AAA using the MBM combined with CFD simulation were obtained.

In addition, we also reconstructed the rigid model and performed the rigid wall simulation, the size of the rigid model was set to be equal to the mean size of the models at 21-time points. We analyzed the differences between the hemodynamics results by performing these two simulations. In general, this paper explored the influence of *in vivo* deformation of the abdominal aorta on hemodynamics and evaluated the formation risk of thrombosis in AAA.

## 2 Materials and methods

### 2.1 Image data acquisition

A 61-year-old male patient with a maximum AAA diameter of 32.99 mm was selected for the acquisition of 4D CT image data. All imaging data were obtained from the Department of Vascular Surgery, Zhongshan Hospital, Fudan University, and permission were obtained from the ethics committee.

**FIGURE 1:**
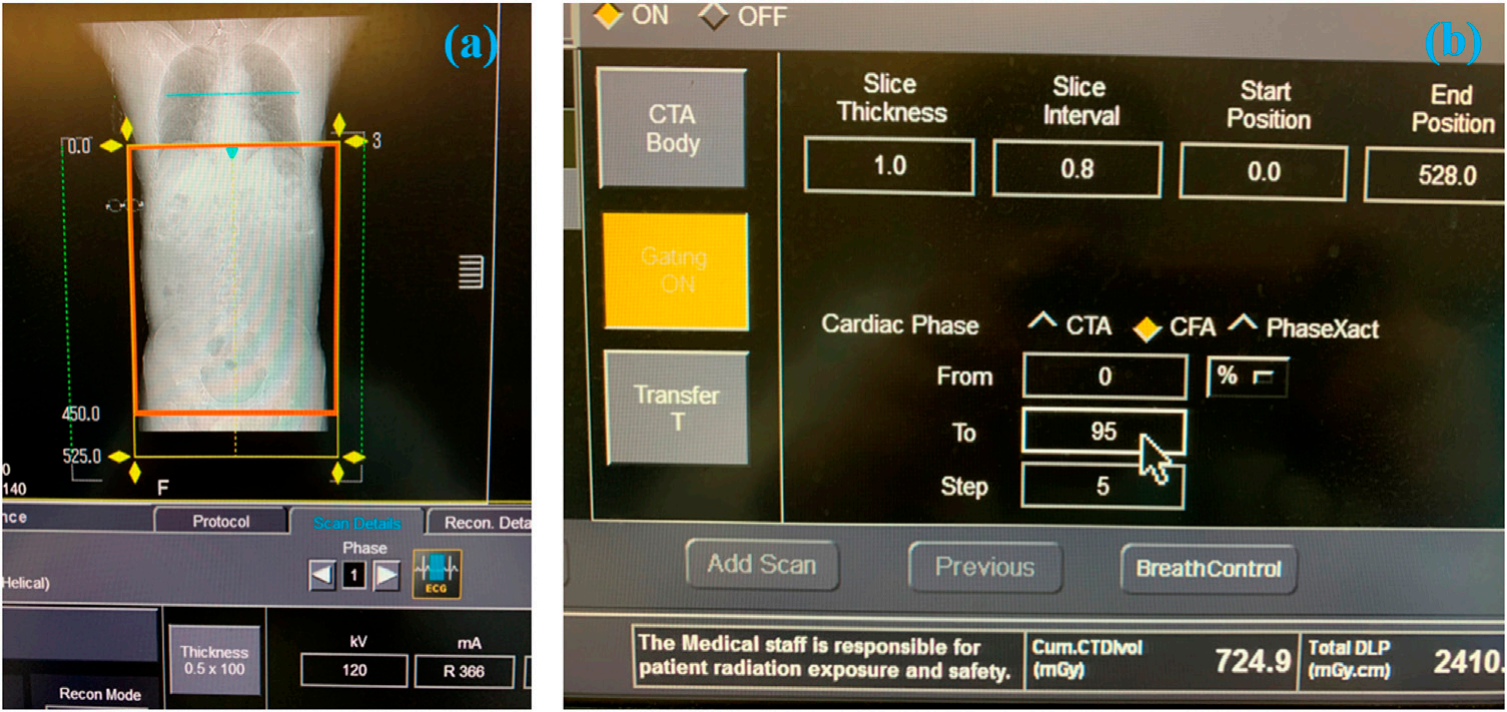
The process of obtaining the 4D CT image data. **(A)** The selected scanning range of AAA. **(B)** Parameters setting during scanning.

The CTA data was obtained by using a 320-row multidetector CT scanner (Aquilion ONE, Toshiba Medical Systems, Irvine, CA, United States). The view field ranged from at least 5 cm proximal to the celiac trunk and the femoral artery bifurcation for each set of CTA data, as shown in Figure 1A.

When scanning the patient, the slice thickness of CTA was 1.0 mm, and the patient was scanned every 5% cardiac cycle, as shown in Figure 1B. The original in-plane resolution matrixes were 512 × 512 (the spatial resolution was 1.12 mm × 1.12 mm), which was low and cannot be used for model reconstruction. By using the B-Spline algorithm, which can be used to interpolate and sharpen the original image data, we obtained the 1024 × 1024 (the spatial resolution was 0.56 mm × 0.56 mm) in-plane resolution matrixes at 21-time points. The deep learning method based on the U-net technique was used for automatic segmentation and to suppress the signal corresponding to the tissue surrounding the aorta (Supported by Shenzhen Rui Xin Intelligent Medical Technology Co., Ltd.). The image of 21 time-instants in one cardiac cycle was stored in DICOM format, which was described in Module 1 of the Supplementary Material.

### 2.2 Model reconstruction

**FIGURE 2:**
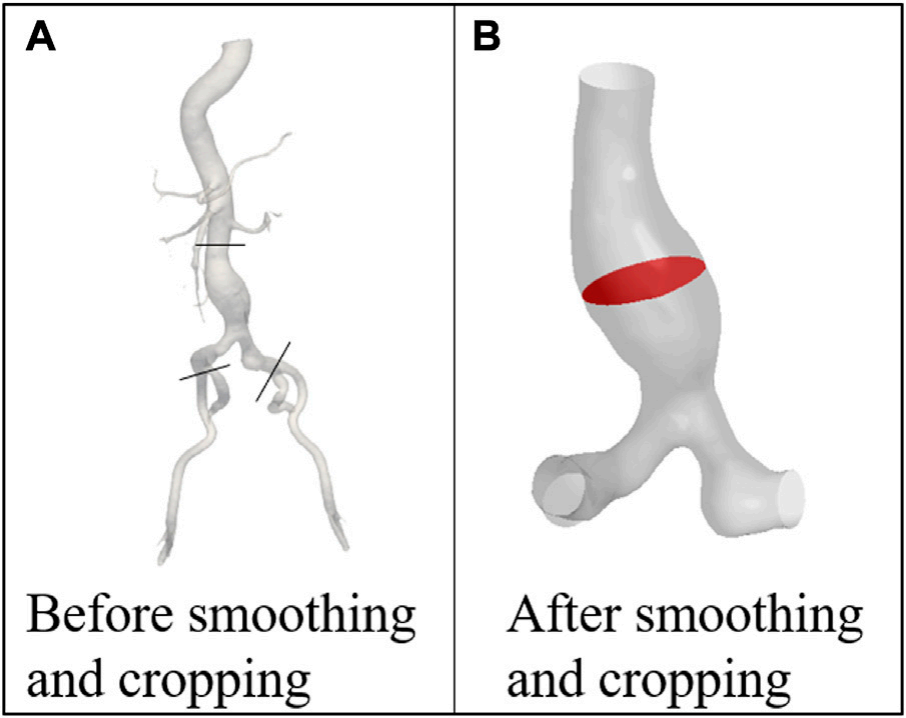
Models of the patient **(A)** before and **(B)** after smoothing and cropping. POI is marked with red color.

The 4D CT image data was imported into the open-source software: SimVascular 2018 (Stanford University, United States) for preliminary smoothing (Lan et al., 2018), as shown in Figure 2A. Since AAAs tend to occur in the infrarenal (IR) abdominal aorta region (Amirbekian et al., 2009), the abdominal aorta and iliac arteries under the renal arteries was selected as the computational domain (Qiu et al., 2018), as shown in Figure 2B. Models at 21-time points were smoothed and cropped in Geomagic Studio 2013 (Raindrop Corporation, United States). The model of the first phase was chosen as the model of the initial moment when using the MBM.

The surface with the largest cross-sectional area perpendicular to the centerline of the AAA was chosen as the plane of interest (POI), which was marked with red color, as shown in Figure 2B.

The effect of the area change ratio due to vessel wall deformation (Eq. 1), the differences between the maximum and minimum areas of POI before and after smoothing (Eq. 2), and the difference in the area change ratio due to the smoothing treatment (Eq. 3) were calculated to check whether the errors caused by smoothing affect the accuracy of simulation and the results are summarized in Table 1.

$$ratio=\frac{A_{\max}-A_{\min}}{A_{\max}}*100\%$$ (1)

$${Di\mathrm{ff}erence}_{A}=\frac{A_{\mathrm{original}}-A_{\mathrm{smoothed}}}{A_{\mathrm{original}}}*100\%$$ (2)

$${Di\mathrm{ff}erence}_{\mathrm{ratio}}=\left\{{ratio}_{\mathrm{smoothed}}\left(\max.\min\right)-{ratio}_{\mathrm{original}}\left(\max,\min\right)\right\}*100\%$$ (3)

where the *A* *_max_*, *A* *_min_* represent the maximum and minimum areas of POI, *A*_*original*_ and *A*_*smoothed*_ represent the POI area values of the original and the smoothed models.

**TABLE 1:** The difference between the maximum and minimum POI area in one cardiac cycle before and after smoothing.

	*A* *_max_* (*mm* ^2^)	*A* *_min_* (*mm* ^2^)	Ratio (%)
Before smoothing	910.32	887.32	2.53
After smoothing	901.43	876.37	2.78
Difference (%)	0.98	1.23	0.25

As shown in Table 1, the differences between the maximum and minimum areas of POI are both less than 3%. The differences in area change are less than 1.5% and the area change rate is only 0.25% before and after smoothing. It indicates that the error caused by the smooth operation is small and would not affect the numerical simulation accuracy.

### 2.3 Mesh generation

**FIGURE 3:**
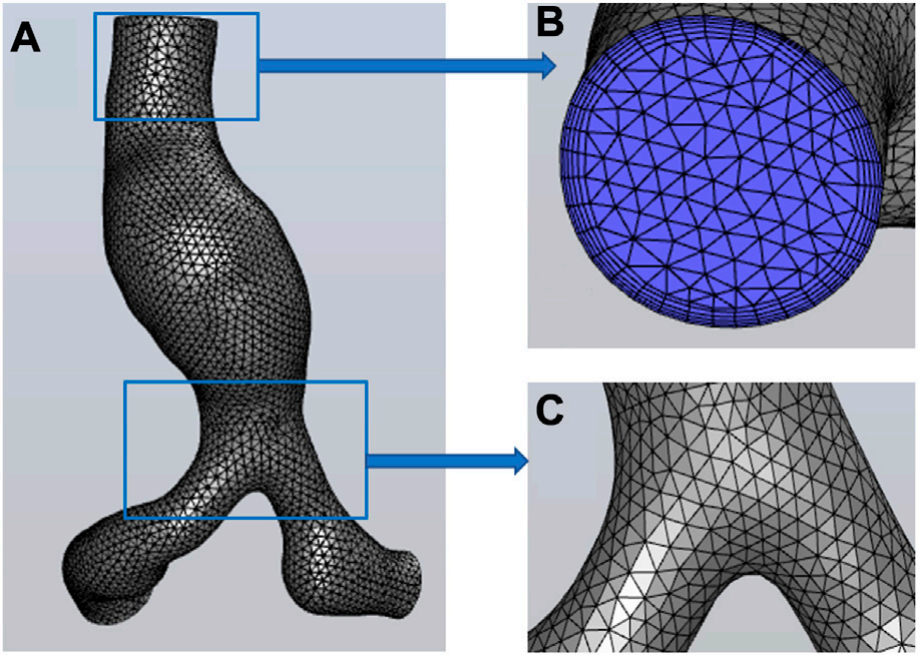
Mesh information. **(A)** the global mesh information, **(B)** the mesh at the inlet and the boundary layer mesh, **(C)** the mesh at the bifurcation of iliac arteries.

The tetrahedral mesh was generated in HyperMesh 14.0 (Altair, United States). The global mesh size of the model at the initial time was set as 0.3 cm, and the wedge-shaped boundary layer mesh was generated in the fluid domain. After the grid independence test (the process is described in Module 2 of the Supplementary Material), the mesh of the initial model is shown in Figure 3. The node number and elements of the fluid mesh, and node number of the surface mesh of the vessel wall at the initial moment were 30,750, 89,022, and 4,917, respectively.

Global mesh densification was performed on models at other 20 time-instants, with the mesh size set as 0.02 cm. The node number of the vessel wall for the subsequent 20 time-instants was 144,650 ± 10,025 (mean ± SD). The process was described detailed in Module 3 of the Supplementary Material. Note that these surface meshes are not involved in computational fluid dynamics simulations, but only store coordinate information of surface nodes at different time points.

### 2.4 Calculation of nodal displacement at different time-instants

**FIGURE 4:**
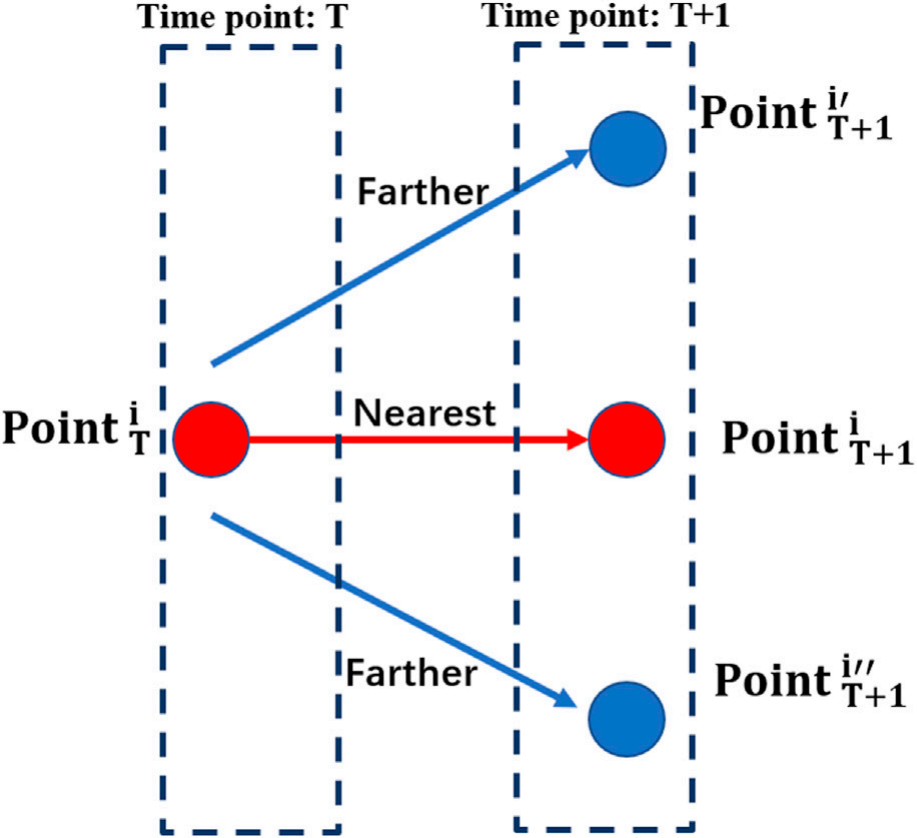
Node displacement calculation between two adjacent time-instants.

As described in Section 2.3, the surface mesh of models was densified at the next 20 time-instants to provide more optional nodes for the grid nodes to move from the current time point to the next (as shown in Figure 4), which can ensure that each node at the current moment could move to its nearest node at the next moment.

As shown in Figure 4, $Point_{T}^{i}$ represents the *i*th node at the current moment, $Point_{T+1,}^{i}\;Point_{T+1}^{i^{\prime }}$ and $Point_{T+1}^{i^{\prime \prime }}$ represent the nodes that could be chosen for $Point_{T}^{i}$ at next moment. It is obvious that $Point_{T+1}^{i}$ is closest to $Point_{T}^{i}$ , and other nodes which are not selected will be deleted.

Matlab R2020b (MathWorks, United States) was used to calculate the coordinate information of nodes at different time-instants. As shown in Figure 4, the nodes’ displacements at different time-instants were calculated according to the minimum distance principle (Vahidkhah et al., 2017). For two adjacent time-instants, the distance of different nodes and the coordinates at the next moment were expressed as Eqs 4, 5:

$$d^{i}=\min|X_{T+1}^{N}-X_{T}^{i}|$$ (4)

$$\left\{\begin{array}{c} x_{T+1}^{i}=x_{T}^{i}+dx^{i}\\ y_{T+1}^{i}=y_{T}^{i}+dy^{i}\\ z_{T+1}^{i}=z_{T}^{i}+dz^{i} \end{array}\right.$$ (5)

where $X_{T}^{i}$ represents the coordinate of the *i*th node at time point T, $X_{T+1}^{N}$ represent the coordinates of all nodes at the moment T+1, and *d* ^*i*^ represents the minimum distance value between the *i*th grid node at moment T and all grid nodes at time phase T+1. Eq. 5 indicates that the coordinates of the next moment in three directions are obtained by adding the coordinates of the current moment to the corresponding distances moved in each of the three directions.

Afterward, the grid node coordinates were interpolated for 21 time-instants [so that the Courant number was less than 1 (Khalafvand et al., 2017)], and 80-time steps were generated for each interval of the 21-time-instants, resulting in 1600 (=20 0180) time steps for one cardiac cycle of computation. The generated coordinate files of each grid node were invoked by the user-defined function (UDF) in Fluent 2019 R3 (ANSYS, Canonsburg, PA, United States). To avoid non-convergence of calculation due to excessive mesh distortion, a remeshing technique was used in Fluent to regenerate new surface mesh as well as meshes in the fluid domain when the maximum mesh skewness was greater than 0.7, the process of performing the MB simulation was also described detailed in Module 4 of the Supplementary Material.

### 2.5 Boundary conditions

**FIGURE 5:**
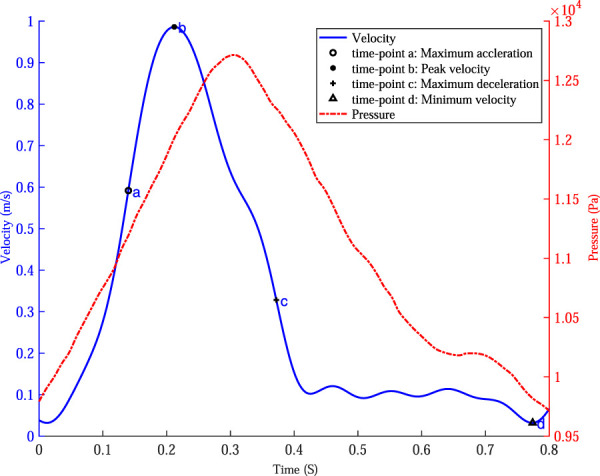
The velocity waveform at the inlet of the abdominal aorta and the pressure waveform at the outlets of the iliac arteries and four specific time points were marked on the velocity profile.

The boundary conditions were all derived from another volunteer, the velocity waveform at the inlet of the IR aorta was measured by 2D phase-contrast magnetic resonance imaging (PC-MRI, and the magnetic resonance System is 3.0 T Discovery MR750, GE Medical System, United States) technique, and the pressure waveform at the outlets of the iliac arteries was measured by the pressure guidewire, as shown in Figure 5.

The UDF function in Fluent was used to specify the velocity of each node at the aorta inlet and specify the pressure waveform of the iliac artery outlets.

Based on the maximum velocity waveform at the inlet of the IR aorta, four specific time points were selected to analyze the hemodynamics in AAA: time of maximum acceleration of velocity (time-point a, 0.14 s), time of maximum deceleration of velocity (time-point c, 0.37 s), time of maximum velocity (time-point b, 0.21 s) and time of minimum velocity (time-point d, 0.78 s).

### 2.6 Solving settings

In the numerical simulation based on MBM, Arbitrary Lagrangian-Eulerian (ALE) method was used to solve the continuity and momentum equation, as shown in Eqs 6, 7:

$$\frac{\partial }{\partial t}\int_{V}\rho dV+\int_{S}\rho \left(\vec{v}-{\vec{v}}_{b}\right)\cdot \vec{n}dS=0$$ (6)

$$\int_{V}\frac{\partial }{\partial t}\left(\rho \vec{v}\right)dV+\int_{S}\rho \left(\vec{v}-{\vec{v}}_{b}\right)\cdot \vec{n}dS=-\int_{S}pI\cdot \vec{n}dS+\int_{S}\tau \cdot \vec{n}dS$$ (7)

where $\vec{v}$ represents the velocity vector of blood, ${\vec{v}}_{b}$ represents the velocity vector of the grid nodes of the vessel wall, $\vec{n}$ represents the normal vector, *ρ* represents the blood density, *p* represents the pressure, **I** represents the unit tensor, and ***τ*** represents the viscous stress tensor. When assuming the vessel wall as rigid, the continuity equation and the Navier-Stokes equation could be simplified into Eqs 8, 9:

$$\frac{\partial }{\partial t}\int_{V}\rho dV+\int_{S}\rho \left(\vec{v}\right)\cdot \vec{n}dS=0$$ (8)

$$\int_{V}\frac{\partial }{\partial t}\left(\rho \vec{v}\right)dV+\int_{S}\rho \left(\vec{v}\right)\cdot \vec{n}dS=-\int_{S}pI\cdot \vec{n}dS+\int_{S}\tau \cdot \vec{n}dS$$ (9)

The blood was assumed as an incompressible Newtonian fluid with a density of 1060 *kg*/*m* ^3^, and the viscosity was set as 0.0035Pa ⋅ *s*. No-slip and no-flux conditions were applied at the vessel wall. The pressure implicit with the splitting of operators (PISO) algorithm was used to solve the continuity and the momentum equations to obtain velocity and pressure. The second-order upwind scheme was used to discrete the control equations at grid nodes of the fluid domain (Khalafvand et al., 2017), the laminar solver was used for the Reynolds number is small and fluid in the lumen of the vessel does not develop turbulence (Peng et al., 2022). The cardiac cycle was0.8 s, the time step size was 0.0005 s, at the end of each time step, the surface mesh and the volume mesh of the fluid domain were remeshed. The residual of continuity and velocity in three directions were set as 1e-4 and each time step is iterated 50 times to ensure that the results converge. The numerical simulations were performed for cardiac cycles. The workstation AMD Ryzen Thread Ripper 3990X 64-core Processor was used. The time requirement was 36 h when performing the MB simulation and 4 h when performing the RW simulation, it should be noted that the size of the rigid model was set to be equal to the mean size of the models at 21-time points so that the comparison could more clearly demonstrate the effects of wall deformation. The TAWSS, OSI, and relative residence time (RRT) were calculated and compared based on the WSS obtained from the simulation results, as shown in Eq. 10 (Peng et al., 2022):

$$\left\{\begin{array}{c} TAWSS=\frac{1}{T}\int |wss|dt\\ OSI=\frac{1}{2}\left\{1-\frac{\left|\int_{0}^{T}wssdt\right|}{\int_{0}^{t}|wss|dt}\right\}\\ RRT=\frac{1}{\frac{1}{T}|\int_{0}^{T}wssdt|} \end{array}\right.$$ (10)

## 3 Results

The simulation results of the last cardiac cycle obtained using both methods were post-processed in CFD Post 2019 R3 (ANSYS, Canonsburg, PA, United States).

### 3.1 Verification of boundary continuity

**FIGURE 6:**
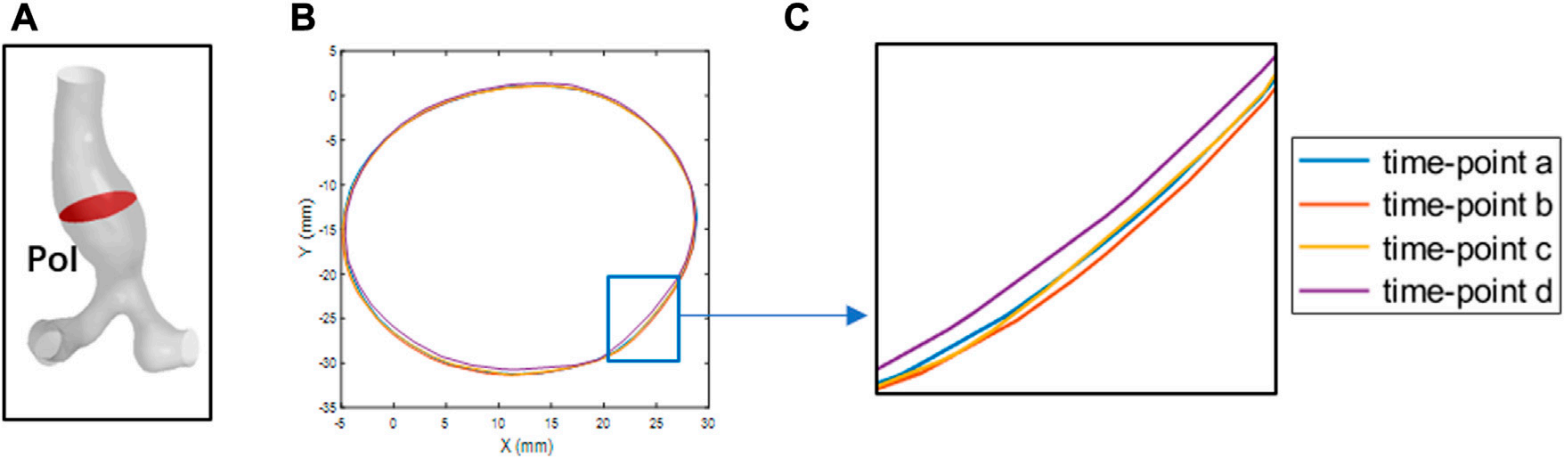
The POI at four-time points is outlined. **(A)** the POI of abdominal aortic aneurysms. **(B)** the boundary contours at the four specific time points **(C)** local magnification of **(B)**.

To verify the boundary continuity when performing the MB simulation, the coordinate information of the POI nodes at four specific time points (time point a to time point d) was derived. Matlab was used to extract the edge contours of the POI for observation, as shown in Figure 6. As shown in Figures 6B, C, the POI contours are continuous and undistorted at four specific time points, indicating that the vessel wall of the abdominal aorta is free of malformed bulges and depressions during the simulation, in line with the requirements for computational quality.

In addition, the overall deformation information was shown in AAA.gif of the Supplementary Material.

### 3.2 Flow fields

**FIGURE 7:**
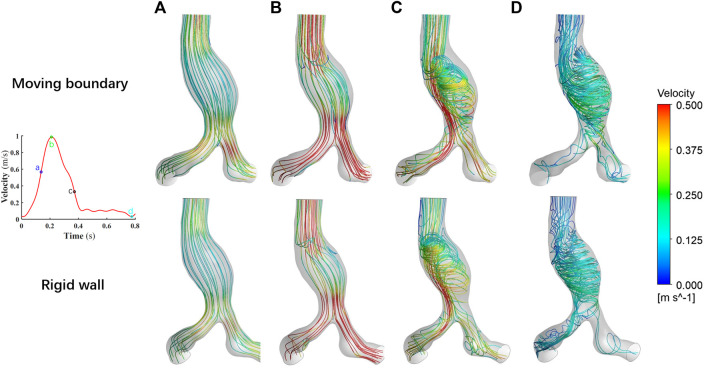
Comparison of the flow fields at four specific time points by using two methods. **(A)** Time moment of maximum acceleration of velocity, **(B)** Time moment of maximum velocity, **(C)** Time moment of maximum deceleration of velocity and **(D)** Time moment of minimum velocity.

The results of the flow field simulated by these two methods are visualized in Figure 7. We narrowed the range of color bar (change the upper limit of velocity into 0–0.5 m/s, i.e.) to make the colors of streamlines more distinct. Overall, the spatial distributions of the flow field at the four specific time points are consistent.

As the blood flow accelerated from time point a to time point b, the flow field within the vessel is stable and no vortex is formed. During this process, the viscous force effect of the blood has little influence on the flow field, while the effect of inertial force is relatively significant. The acceleration effect of the flow is greater than the convective deceleration effect caused by the widening of the aorta. The flow field manifests itself mainly as an ordered laminar flow. However, during the deceleration of the blood at the inlet, the viscous effect of the blood is obvious. The flow state of the blood in AAA gradually becomes disordered. At time point d, the blood flow attaches to the bulging aneurysm wall, and the vortex and secondary flow form within the AAA. In the area near the AAA wall, the recirculation region appears.

### 3.3 Wall shear stress

**FIGURE 8:**
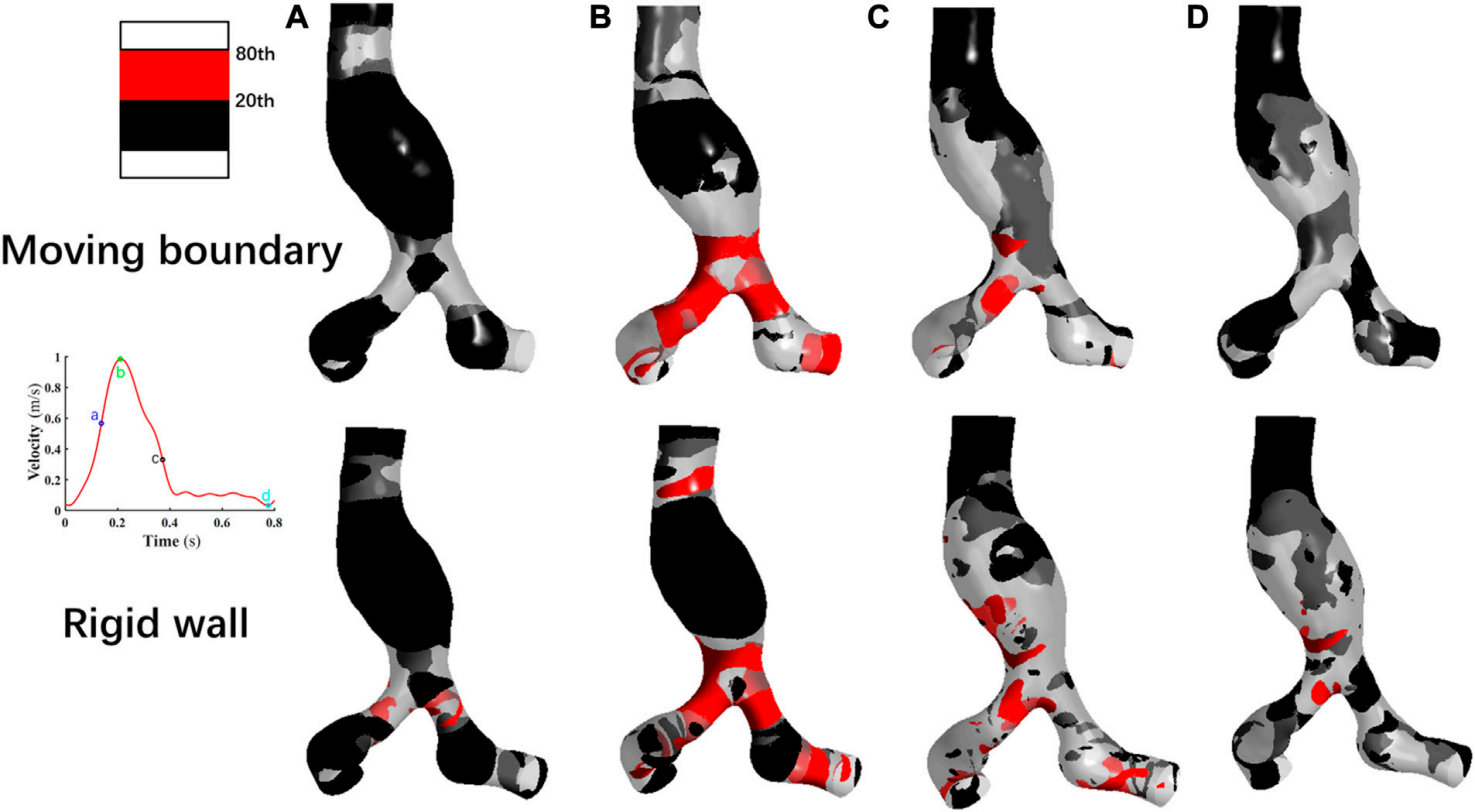
Comparison of the wall shear stress distribution at four specific time points by using two methods. **(A)**: Time moment of maximum acceleration of velocity, **(B)**: Time moment of maximum velocity, **(C)**: Time moment of maximum deceleration of velocity and **(D)**: Time moment of minimum velocity.

The distribution of the WSS on the vessel wall is shown in Figure 8. Overall, the WSS distributions at the four corresponding specific time points are consistent using these two methods. In the region close to the POI, the low WSS values (20th percentile values) appear and indicated a weak shearing effect of blood on the AAA. As the inlet blood velocity increases, the high WSS values(80th percentile values) gradually appears at the bifurcation of the abdominal aorta and iliac arteries, which may lead to endothelial cell damage and destruction (Liu et al., 2014). As the inlet blood flow velocity decreases, the regions with high WSS values gradually reduce. At time point c, the high WSS based on MB simulation is concentrated in the abdominal aortic bifurcation and the iliac arteries, and the high WSS obtained by assuming the vessel wall as rigid occurs in these locations as well as in localized regions of the aneurysm wall. At time point d, most of the WSS values obtained by using the MBM were lower than 20th percentile values. When performing the RW simulation, a small number of high WSS values still appears in parts of the abdominal aortic bifurcation, in the region where the iliac arteries join the AAA and in the distal neck of the AAA.

Notably, the distribution region of high WSS obtained by assuming the vessel wall as rigid is more extensive than that of MBM. The ratios of high WSS area to the whole vessel wall are 0.54%, 14.33%, 8.57%, and 0.15% at four specific time points by using MBM, and these ratios obtained from the RW simulation are 2.41%, 28.62%, 15.32%, and 2.18%, respectively.

### 3.4 TAWSS, OSI, RRT

TAWSS, OSI and RRT were calculated using Eq. 10 to assess the blood stagnation status and to further predict the risk of thrombus formation.

**FIGURE 9:**
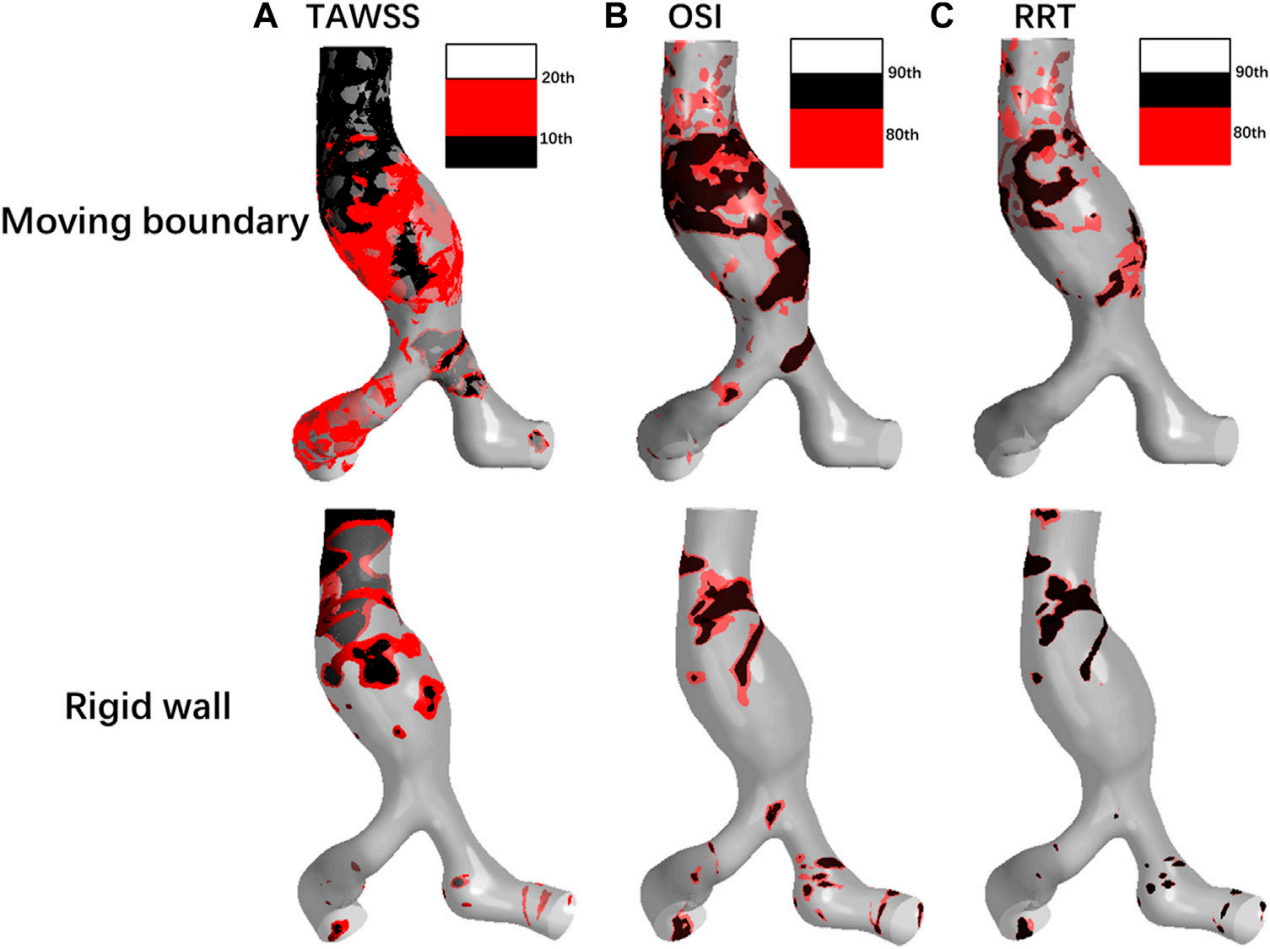
Comparison of **(A)** TAWSS, **(B)** OSI, and **(C)** RRT distributions by using two methods.

As shown in Figure 9A, the TAWSS simulated by using MBM fluctuates slightly throughout the vessel wall, with distribution in most regions of less than 10th percentile values. In contrast, the TAWSS obtained from the RW simulation differs significantly across regions. Low TAWSS is mainly concentrated on the aneurysm wall, but in the downstream region of the AAA neck and the iliac artery bifurcation, TAWSS are larger than 20th values.

Besides, the ratios of the area where the low TAWSS located to the entire vessel wall area are 37.85% and 11.71% by using MBM and assuming the vessel wall as rigid, respectively.

As shown in Figure 9B, the high OSI (larger than 80th percentile values) obtained by using MB simulation is distributed over most of the aneurysm wall, AAA’s neck, and the iliac arteries, whereas the higher OSI obtained by assuming rigid vessel wall is relatively concentrated in the proximal neck of AAA and the bifurcation of the iliac arteries. In general, the region of high OSI obtained by using MBM is more widespread. Moreover, the percentage of the area with high OSI to the whole vessel wall obtained by using MBM is higher than that obtained from the RW simulation (69.32% and 21.72%, respectively). It implies the direction of the shear stress has a faster change frequency when using the MBM, since the direction of the blood velocity near the aorta changes constantly when applying the no-slip conditions. High OSI produces an oscillatory effect on the vessel wall and reflects a higher risk of damage to aorta compared to the rigid wall.

RRT obtained using the two methods are shown in Figure 9C. The high RRT (larger than 80th percentile values) obtained using MBM are distributed in the region of the proximal neck of AAA and adjacent to the POI. For results obtained from RW simulation, high RRT are relatively concentrated in the region of aneurysms’ proximal neck. High RRT obtained by using MBM spreads more widely on the vessel wall than those obtained from RW simulation. The ratios of the area of the region where high RRT locates to the whole vessel wall area obtained from these two methods are 24.12% and 6.37%, respectively. Moreover, the distribution regions with high RRT are generally consistent with the distribution regions of high OSI.

Furthermore, when comparing the flow field and RRT distribution obtained by using MBM, high RRT tends to be concentrated near the recirculation region of the flow field. When the blood flow velocity is low and the streamline takes on a spiral shape, the substances in the blood may stagnate for a long time near the aneurysm wall. Leukocytes, platelets and other substances may have stronger migration and adhesion effects in such a hemodynamic environment. This suggests that the effect of prolonged high RRT and recirculation region may increase the risk of thrombosis near the AAA.

To quantitatively compare the TAWSS, OSI, and RRT of each case simulated by these two methods, the mean values of the three parameters on the vessel wall were calculated, which are summarized in Table 2. The mean value of TAWSS obtained from the RW simulation is 18.18% higher, while the mean OSI value and mean RRT value are 42.31% and 11.61% lower than the results obtained from the MB simulation, respectively. It may indicate that results obtained from the MBM reflect a higher effect of blood stagnation.

**TABLE 2:** Comparison of the mean value of three parameters on the vessel wall by using two methods.

Methods	TAWSS(Pa)	OSI	RRT(Pa^−1^)
Moving Boundary	1.35	0.26	2.24
Rigid wall	1.65	0.15	1.98

### 3.5 The comparison between the MB simulation results and follow-up image data

**FIGURE 10:**
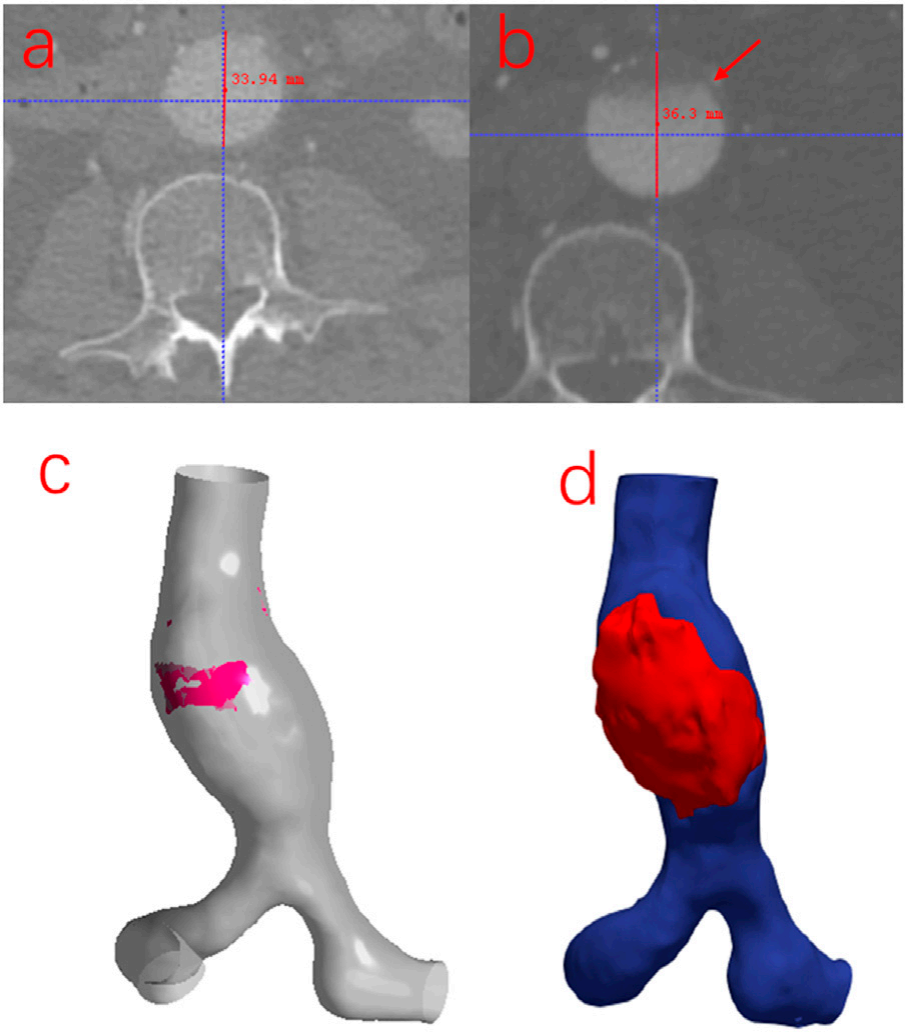
**(A)** The initial cross-section of the maximum diameter of AAA. **(B)** The cross-section of the maximum diameter of AAA was obtained from the follow-up image data. **(C)** The overlapping region of low TAWSS, high OSI, and high RRT obtained from the MB simulation: is marked in red color. **(D)** The location of the thrombus was reconstructed from the follow-up image data.

To investigate whether there is an association between the simulation results when using the MBM and the risk of thrombosis and the growth of AAA, the follow-up data from the same patient was collected after 2 years in this study. As shown in Figure 10, the ILT forms in the region near the POI but the maximum diameter of the AAA increases by only 2.36 mm. There is an overlapping region with low TAWSS, high OSI and high RRT when using the MBM. (Notably, there is no overlapping region when performing the RW simulation.) Although the ratio of the area of the overlapping region to the entire vessel wall is only 4.92%, the thrombus almost occupies the anterior wall of the aneurysm near the overlapping region after 2 years. It indicates a higher likelihood of thrombosis when all three factors (low TAWSS, high OSI and high RRT) mentioned above work together, and the ILT may prevent the further growth of AAA.

## 4 Discussion

The combination of CFD and medical imaging data has been widely used for hemodynamics simulation in the clinical assessment of cardiovascular disease in patients. However, the traditional RW simulation does not consider the deformation of the vessel wall, which does not conform to the actual physiological conditions (Vergara et al., 2017). Although the FSI method can simulate the interaction of the vessel wall with blood, the results may differ from the real hemodynamics due to the difficulty of obtaining patient-specific vessel wall material parameters quickly and accurately (Shamloo et al., 2020).

In this paper, the 21-time phases models in one cardiac cycle of one AAA patient were reconstructed and the mesh was generated. After that, the spatial coordinates of each mesh node at different time-instants were calculated, then the UDF was used to control the displacement of each node without considering the material properties of the vessel wall. We also assumed the vessel wall as rigid for another simulation, and the differences between the results of these two simulations were compared to analyze the influence of the vessel wall deformation on hemodynamics in AAA.

The spatial distribution of flow fields simulated by using the MBM and assuming the vessel wall as rigid is similar. Notably, at time-point d, the flow field near the AAA is very disorganized with a large number of vortexes. It may indicate that aneurysm formation may have undesirable effects on laminar flow patterns of blood, which has the potential to damage the endothelial cells (Hsu et al., 2001).

The TAWSS was calculated as the tangential component of traction on the vessel wall through the cardiac cycle. Zambrano et al. (2016) found that the ILT accumulated in areas of low TAWSS. Once ILT initially gathered near the lumen wall, it enhanced the effect of aggregation of new thrombus at regions where TAWSS was low. In regions of low TAWSS, red blood cells, platelets, and other substances within the blood were transported in a smaller area near the wall, which indicated a stronger stagnation effect in the near-wall region (Bilgi and Atalık, 2019).

The results of OSI, and RRT distribution on the vessel wall are quite different using these two methods. We speculated these parameters related to WSS strongly correlated with the deformation of the vessel wall, according to Gallo et al., we selected the 10th and 20th percentile for TAWSS, 80th and 90th values for OSI and RRT, which can provide a more complete and proper analysis of the difference between the rigid wall and moving boundary simulations.

High OSI indicates that during the movement of the blood vessel wall, the direction changes the frequency of the shear stress near the vessel wall is fast. This may be because when using the no-slip boundary condition, the blood flow velocity at the near-wall surface changes continuously during the cardiac cycle, as does the vessel wall. High OSI damage to the endothelial cells is serious. Prolonged high OSI may exacerbate changes in the orientation of endothelial cell alignment in these regions (Bilgi and Atalık, 2019), cause inflammation in the intima layer of the vessel wall and degeneration of elastin in the media layer of aorta, and lead to an increased risk of thrombus formation (Meng et al., 2014; Soldozy et al., 2019).

Besides, the high/low OSI is usually accompanied by high/low RRT (Liu et al., 2021). Since high OSI causes damage to the endothelium, which is accompanied by the release of clotting factors, platelets, lipids and other substances, the blood undergoes a deposition effect and adheres to the vessel wall (Chen et al., 2018). A high RRT indicates the blood stays in the low-velocity recirculation region of the aneurysm for a long time, prolongs the contact time between blood substances and the low-velocity layer near the vessel wall, and creates conditions for the migration and aggregation of platelet white blood cells and coagulation factors into the aneurysm wall (Zambrano et al., 2016; Kelsey et al., 2017; Yeow and Leo, 2018).

In general, the ILT formation is associated with low TAWSS, high OSI, and high RRT, which may cause damage to the intima of the vessel wall in AAA, promote the inflammatory reaction on the vessel wall, and release the coagulation factors and lead to the formation and growth of thrombus.

The diameter of the maximum cross-section of AAA only increases by 2.36 mm, while there is a large amount of ILT formation in the anterior wall of the AAA. The ILT effectively reduced wall stress in AAAs, which provided AAAs from rupture (Lindquist Liljeqvist et al., 2020). A large strain constitutive relation into patient-specific AAA simulations and demonstrated that the presence of the ILT can significantly reduce wall stress by up to 38% (Piechota-Polanczyk et al., 2015). Our results also suggested to some extent that ILT could prevent AAA growth and rupture, so that the diameter of the AAA does not increase significantly.

There is still considerable potential for improvements in this study. We used the physiological data of the previous volunteer as the boundary conditions for numerical simulation. The PCMRI and ultrasound techniques can be used to set individual boundary conditions for each patient (Peng et al., 2022). In addition, due to the limitation of spatial resolution, it is difficult to accurately obtain the deformation information of the visceral arteries. Therefore, this paper only selected the AAA and iliac arteries to reconstruct patient-specific models. The visceral arteries (such as the renal arteries, and the trunk celiac artery) also needed to be considered in the numerical simulation. Assuming the blood as Newtonian fluid and ignored its viscoelastic was also the limitation of our study. We also consider collecting 4D CT image data of more AAA patients for MB simulation.

In general, this paper has demonstrated the feasibility of applying the MBM to the hemodynamics simulation within AAA. It could describe the vessel wall deformation, and provide effective reference information for clinical evaluation of the ILT formation risk in AAA.

## 5 Conclusion

We simulated the hemodynamics in this paper under the *in vivo* deformation of vessel wall based on 4D CT medical image data, proved the feasibility of the MBM, and compared the simulation results with those assuming a rigid vessel wall. The overall spatial distributions of flow fields and WSS by using the MBM and assuming a rigid vessel wall are similar. Besides, deformation of the vessel wall leads to the lower TAWSS, higher OSI and higher RRT distributions. The simulation results obtained from the MBM reflect a higher blood stagnation effect in AAA. And the combination of the MBM with 4D CT image data may provide a new idea for the clinical prediction of the ILT formation risk in AAA.

## Data Availability

The original contributions presented in the study are included in the article/Supplementary Material, further inquiries can be directed to the corresponding authors.
