# Supplementary material for: The study on the impact of AAA wall motion on the hemodynamics based on 4D CT image data
Source: Front Bioeng Biotechnol. 2023 Mar 30;11:1103905. doi: 10.3389/fbioe.2023.1103905 (PMC10098133; doi:10.3389/fbioe.2023.1103905)
Supplement: Supplementary file 5 [file DataSheet1.PDF]

Supplementary Material

1 MODULE 1: U-NET TRAINING METHOD

The peripheral vascular CT image processing software was used to obtain the original DICOM data of peripheral vascular CT. BSpline interpolation algorithm was used to interpolate the medical image data, etc., to achieve the results of sampling twice on each of the three dimensions.

Preprocessing the original medical image data. To improve the accuracy and robustness of deep learning algorithms in segmentation tasks. The preprocessing steps included CT interpolation, whitening and data amplification.

The U-Net based deep learning model algorithm is trained and tested on medical image data to reconstruct a 3D model of blood vessels. The CTA images of AAA are large and the volume needs to be cropped into some small patch size (e.g. 128x128x128) with randomly sampled center points of the patch. During testing, the sampled points are combined into a volume by sequentially speaking the segmentation results of the patches.

For the generated segmentation results, some anomalies or segmentation regions are removed to ensure the robustness of the software algorithm. The specific approach is as follows: the segmented result is processed for the maximum joint region, and the final result is the segmented vessel result.

The specific implementation process is as follows:

Preoperative CTA images were obtained from 100 consecutive patients with an infrarenal AAA who had multidetector CT scanners with 1-mm-thick slices of the entire abdominal aorta and arterial phase intravenous injection of contrast liquid at Zhongshan Hospital of Fudan University (Shanghai, China). The 100 CTAs included in our study came from four different scan manufacturers, 53 from General Electric scanners, 18 from Siemens and Philips scanners, 10 from Toshiba, and 1 from an unknown manufacturer, 53 from General Electric scanners, 18 from Siemens and Philips scanners, 10 from Toshiba, and 1 from an unknown manufacturer.

Images were given in Digital Imaging and Communications in Medicine format providing a matrix of size 512x 512 for each slice. Patients with pararenal or thoracoabdominal aneurysms, associated iliac artery aneurysms, ruptured aneurysms, aortic dissections, and non-contrast CTs were excluded from the analysis. The experimental protocol was approved by the institutional review board, and all patients gave informed consent.

In the experiment, training set of 80 CTAs for a test set of 20 fully segmented CTAs.

2 MODULE 2: THE GRID DEPENDENCY TEST

An appropriate mesh size and elements number not only ensure the accuracy of the calculation but also save computational resources. A grid dependency test was done under five mesh sizes with the same BCs: 0.9, 0.6, 0.3, 0.15, and 0.05 cm, and the maximum WSS value was used as an indicator to measure the grid convergence.

In Figure S1, the first row lists the mesh information under five mesh sizes, the maximum values of WSS under the five mesh sizes are shown in the second row of Figure S1. The constant pressure (80 mmHg) was imposed on the outlet of iliac arteries, and constant velocity (0.35 m/s) was imposed on the inlet of the abdominal aorta.

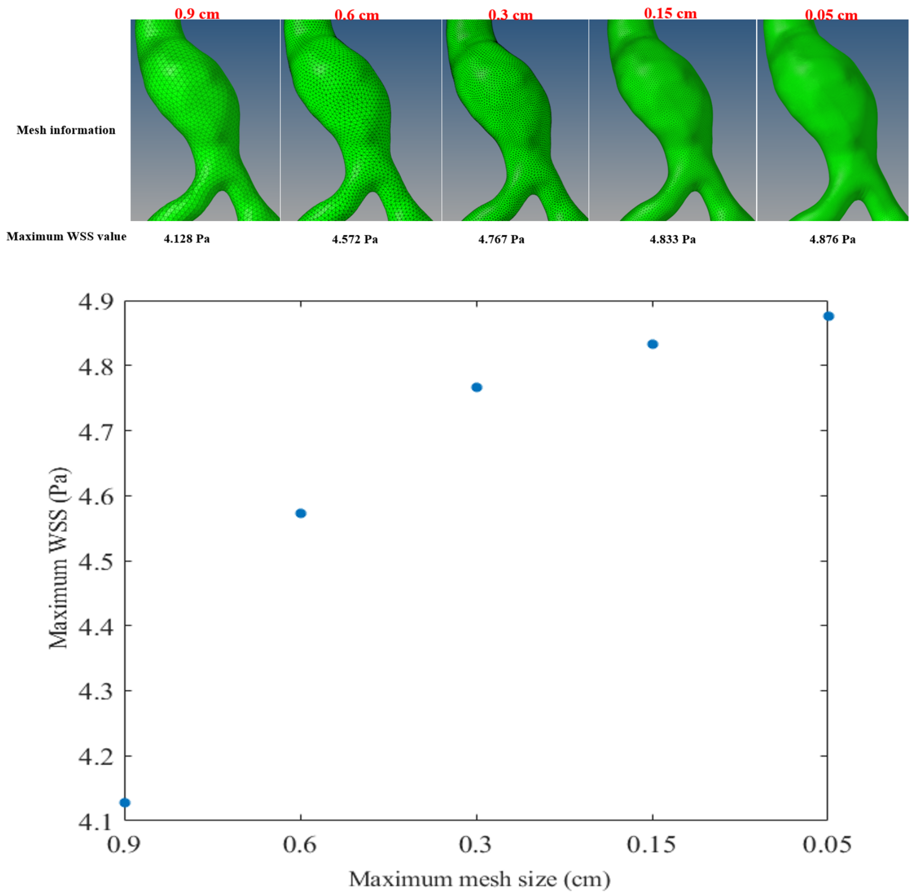

Figure S1: The mesh information, maximum WSS values and the relationship between mesh sizes and maximum WSS values under different mesh sizes.

Mu et al.,[1] found that the grid with less than 5% pressure error was considered acceptable. As shown in the third row of Figure S1, the maximum WSS values corresponding to the mesh size of 0.9 cm, 0.6 cm, and 0.3 cm are 4.128 Pa, 4.572 Pa, and 4.767 Pa, and change rates of WSS corresponding to the two adjacent mesh sizes are 9.71 % and 4.09 %. And the maximum WSS values vary little when the maximum grid size is less than 0.3 cm.

3 MODULE 3: THE PROCESS THAT GENERATES SURFACE MESH WITH HIGHER DENSITY

In section 2.3 of the revised manuscript, we described the process we generate the mesh of the initial moment (time 0, i.e.), and the result is shown in Figure 3 of the revised manuscript, which includes the surface mesh, the volume mesh, and the boundary layer mesh.

Taking the model of time 1 as an example, the original model was imported into Geomagic Studio, as shown in Figure S2(a). After smoothing and cropping, the iliac arteries and the abdominal aorta were saved, as shown in Figure S2(b) (It should be noted that the retention range of the models at other moments should be larger compared to the model at the initial moment). The global mesh densification was performed as shown in Figure S2(c), and the local zooming was performed as shown in Figure S2(d) to make the grid look clearer.

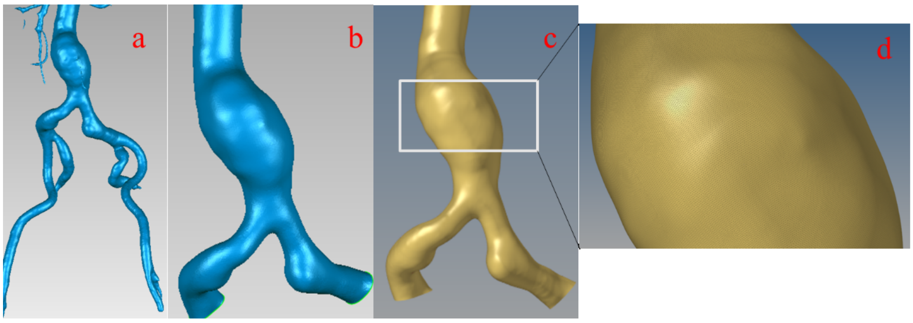

Figure S2: The surface meshes generation process by performing the global mesh densification. (a) The model of time 1 before smoothing and cropping. (b) Model after smoothing and cropping. (c) The mesh size is set as 0.02 cm. (d) Local zooming of the surface mesh.

Similarly, global mesh densification was performed on the models at another 19 moments. Note that these surfaces mesh were not involved in computational fluid dynamics simulations, but stored coordinate information of surface nodes at different time points.

4 MODULE 4: THE PROCESS OF PERFORMING THE MOVING BOUNDARY SIMULATION

The flow chart for moving boundary simulation in combination with CFD is shown in Figure S3. **Steps 1-3 are relatively simple and have been described in the submitted manuscript.** And steps 4-8 are important and we will focus on describing these five steps.

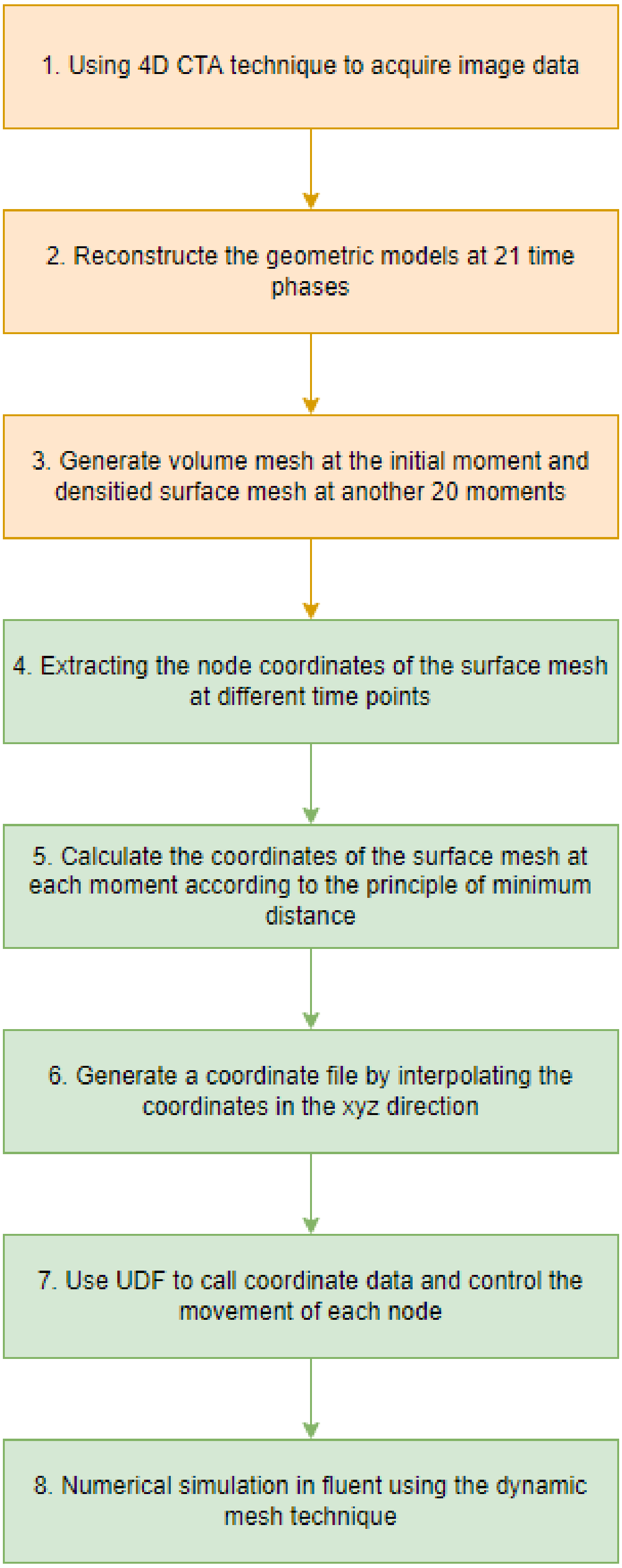

Figure S3: The flow chart of the moving boundary simulation.

4.1 step 4

After generating mesh at 21-time points, we exported the coordinate information of the vessel wall in Fluent 2019 R3, as shown in Figure S4 (to facilitate subsequent calculations, we have standardized the units in the numerical simulation process to the International System of Units).

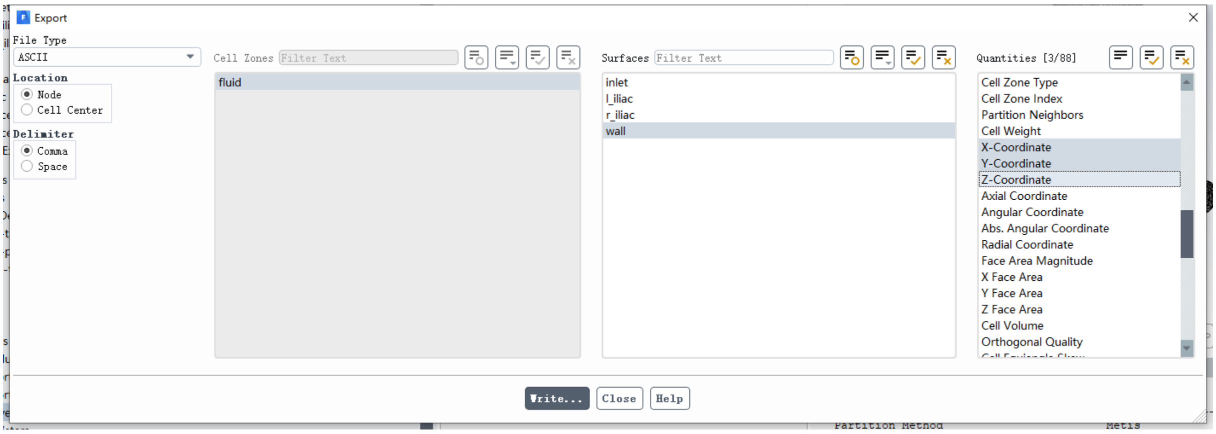

Figure S4: Export the coordinate information of the vessel wall in Fluent 2019R3.

4.2 step 5

In the next step, we calculated the nodal displacement at different time points by using MALAB according to the minimum distance principle, and the pseudocode is shown in Figure S5

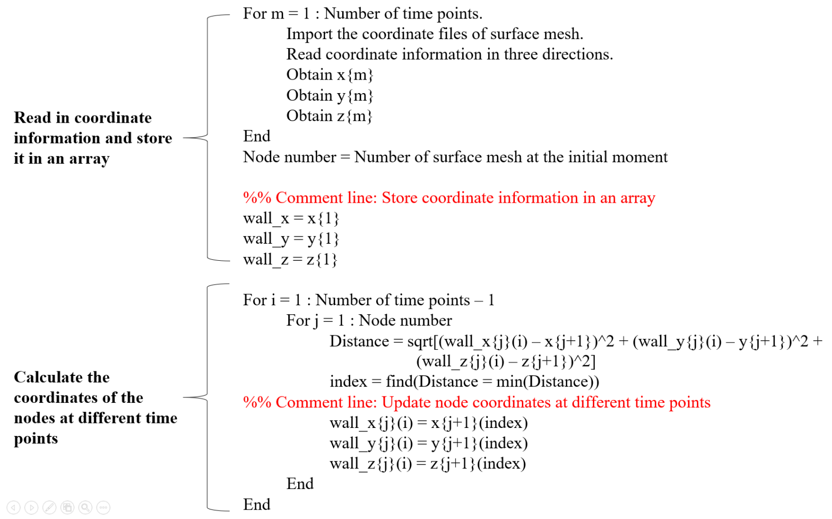

Figure S5: The pseudocode of obtaining the coordinate information at 21 time-points.

4.3 step 6

During the numerical simulation, we need to control the speed of the mesh movement so as not to distort the mesh, which requires the grid not to move too far in a given time step, and the first-order derivative of the displacement for time (velocity, e.g.) needs to be continuous. Therefore, we interpolated

the coordinates of the grid nodes in three directions for 21 moments. And 80-time steps were generated for each interval of the 21-time moments, resulting in 1600 (=20×80) time steps for one cardiac cycle of computation [2].

We kept the values corresponding to the coordinates to 16 decimal places to avoid the effect of the numerical accuracy on results and saved the coordinate information of each node at 1600-time steps for one cardiac cycle.

4.4 step 7

After that, the user-defined function (UDF) was used to call the generated coordinate files, which can be compiled to customize the boundary conditions of the vessel wall[3, 4]. We called the macro DEFINE GRID MOTION in UDF and the pseudocode is shown in Figure S6.

Read coordinate files

```
DEFINE_GRID_MOTION(wall, domain, dt, time, dtime)
{
//Comment line, read coordinate files generated by using MATLAB
File_x = fopen("xx_interpolated.txt", "r")
File_y = fopen("yy_interpolated.txt", "r")
File_z = fopen("zz_interpolated.txt", "r")
for(i=0;i<NN;i++)
  {
    for(j=0;j<NT;j++)
    {
      fscanf(File_x, "%lf", &xx[i][j]);
      fscanf(File_y, "%lf", &yy[i][j]);
      fscanf(File_z, "%lf", &zz[i][j]);
    }
  }
```

Control the movements of grid nodes

```
//Comment line, control the movement of grid nodes
begin_f_loop(f,tf)
{
  f_node_loop(f,tf,n)
  {
    V = F_NODE(f,tf,n)
    if (NODE_POS_NEED_UPDATE(V))
    {
      for(ni=0;ni<NN;ni++)
      {
        for(ti=0;ti<NT;ti++)
        {
          NODE_X(V) = XX[ni][ti];
          NODE_Y(V) = YY[ni][ti];
          NODE_Z(V) = ZZ[ni][ti];
          NODE_POS_UPDATED(V);
          break
        }
      }
    }
  }
end_f_loop(f,tf)
}
```

Figure S6: The process of controlling the movement of each node.

The variables were defined as double to ensure the numerical accuracy. After that, the .c file was built and loaded in Fluent, at this point the compiled wall motion function can be called to control the motion of the mesh nodes on the vessel wall, as shown in Figure S7.

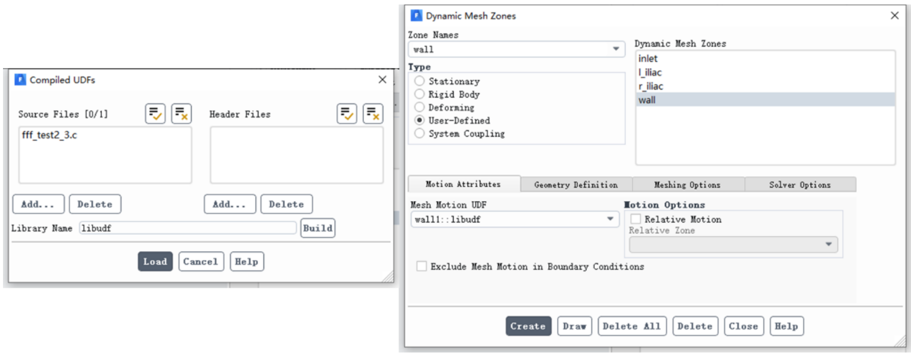

Figure S7: The process that compiles and calls the moving boundary function.

4.5 step 8

It should be noticed that the built-in dynamic mesh technique of fluent was used when using the moving boundary method. According to the fluent tutorial and the experience, the diffusion smoothing method was used and the maximum cell/face skewness was set as 0.7 in the remeshing module, which can avoid non-convergence of calculation due to excessive mesh distortion, as shown in Figure S8.

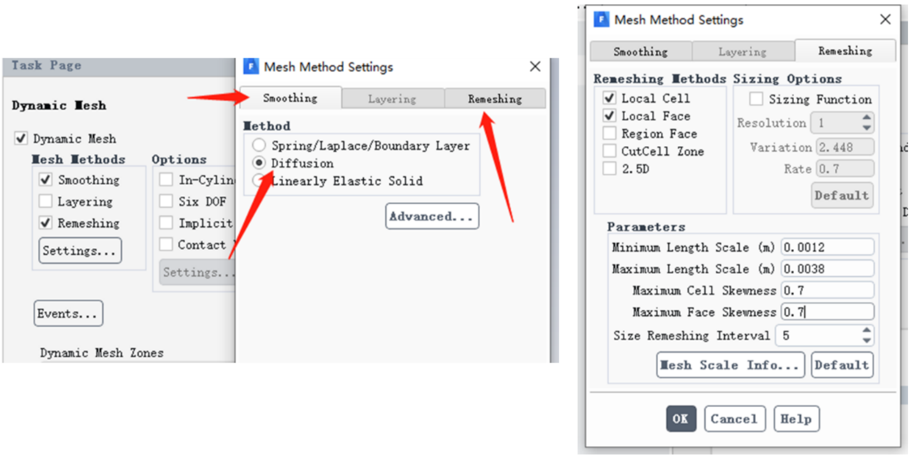

Figure S8: The setting of parameters when using the dynamic mesh technique in Fluent.

The time step number was 1600 and the time step size was 0.0005 s for one cardiac cycle. And the pressure, velocity, and other hemodynamics parameters can be calculated and visualized.

REFERENCES

[1]Zhenxia Mu, Xiaoyu Qiu, Dawei Zhao, Xiaoshuai Li, Minrui Fu, Youjun Liu, Bin Gao, Pengfei Zhao, and Zhenchang Wang. Hemodynamic study on the different therapeutic effects of sswd

resurfacing surgery on patients with pulsatile tinnitus. *Computer methods and programs in biomedicine*, 190:105373, 2020.

[2]Seyed Saeid Khalafvand, Eddie Yin-Kwee Ng, Liang Zhong, and Tin-Kan Hung. Three-dimensional diastolic blood flow in the left ventricle. *Journal of biomechanics*, 50:71–76, 2017.

[3]Fluent. Fluent 14.5 user guide. *Fluent Inc., Lebanon. NH-03766*, 2002.

[4]UDF Manual. Ansys fluent 12.0. *Theory Guide*, 2009.
